# Liver Injury and Cell Survival in Non-Alcoholic Steatohepatitis Regulated by Sex-Based Difference through B Cell Lymphoma 6

**DOI:** 10.3390/cells11233751

**Published:** 2022-11-24

**Authors:** Akihide Kamiya, Kinuyo Ida

**Affiliations:** Department of Molecular Life Sciences, Tokai University School of Medicine, 143 Shimokasuya, Isehara 259-1193, Japan

**Keywords:** non-alcoholic steatohepatitis, non-alcoholic fatty liver disease, inflammation

## Abstract

The liver is a crucial organ for maintaining homeostasis in living organisms and is the center of various metabolic functions. Therefore, abnormal metabolic activity, as in metabolic syndrome, leads to pathological conditions, such as abnormal accumulation of lipids in the liver. Inflammation and cell death are induced by several stresses in the fatty liver, namely steatohepatitis. In recent years, an increase in non-alcoholic steatohepatitis (NASH), which is not dependent on excessive alcohol intake, has become an issue as a major cause of liver cirrhosis and liver cancer. There are several recent findings on functional sex-based differences, NASH, and cell stress and death in the liver. In particular, NASH-induced liver injury and tumorigeneses were suppressed by B cell lymphoma 6, the transcriptional factor regulating sex-based liver functional gene expression. In this review, we discuss cell response to stress and lipotoxicity in NASH and its regulatory mechanisms.

## 1. Introduction

The liver is the largest internal organ and has various functions, such as serum protein synthesis, carbohydrate and lipid metabolism, and detoxification of foreign substances and alcohol. Hepatocytes are the key cells involved in these functions, and express many liver function genes, such as metabolic enzymes. For examples, cytochrome P450 (CYP) is a family of monooxygenase important for steroids, fatty acids and xenobiotics metabolism. CYP is divided into various subfamilies such as CYP1, 2, and 3. Each species expresses its own CYP gene, and there are about 50 types in humans and about 100 types in mice. Lipid metabolism such as fatty acid synthesis and β-oxidation is one of the important liver functions and is regulated by many related enzymes. In addition, lipid metabolism and glucose metabolism are correlated, and the imbalance of these metabolisms causes metabolic syndrome. Sterol regulatory element binding protein (SREBP) and carbohydrate response element binding protein are known as important transcription factors regulating these lipid and glucose metabolic enzymes.

In addition, the liver has a high regenerative capacity; hepatocyte hypertrophy and proliferation are induced in response to liver injury, and liver function is restored during liver regeneration [1]. However, continuous liver injury causes inflammation and extracellular matrix deposition, leading to serious liver diseases, such as cirrhosis and liver cancer. Until recent years, the main causes of chronic liver disease have been alcoholic steatosis, due to excessive alcohol consumption, and viral hepatitis, due to persistent infection with hepatitis B and C viruses (HBV and HCV, respectively). However, with the development of several drugs for HBV and HCV, the number of viral hepatitis cases has decreased, making it possible to eliminate the virus at a considerable rate, especially HCV [2].

In contrast, metabolic syndrome caused by intake of a high-calorie diet and lack of exercise has recently been increasing [3,4]. Non-alcoholic fatty liver disease (NAFLD) consists in the accumulation of lipids, such as triglycerides, in the liver, even though the amount of alcohol consumed is 20 g or less per day in terms of ethanol. These fatty livers do not show any particular pathological symptoms at the stage of simple steatosis and have a good prognosis. However, when NAFLD is combined with several other stressors, non-alcoholic steatohepatitis (NASH), which causes persistent inflammation of the liver, develops and progresses to cirrhosis and liver cancer [5,6]. At first, Day proposed the “two-hit theory”. The cause of NAFLD is obesity associated with metabolic syndrome, dyslipidemia, fat deposition in the liver due to diabetes, etc. as a first hit, which then progresses to NASH due to a second hit, namely inflammatory cytokines, endoplasmic reticulum (ER) stress, and oxidative stress [7]. However, the mechanisms that induce NASH are now considered more complicated. Therefore, a “multiple parallel hit model” involving more stressors has recently been proposed [8]. The pathological condition of the liver and various extracellular stressors are involved, but there are many unclear points regarding the molecular mechanisms that promote steatosis and liver inflammation. In addition, there are no valid therapies for NASH. Therefore, liver transplantation is increasing for the treatment of NASH progression. The establishment of a standard therapy for NASH fibrosis using therapeutic agents to address a new NASH-causing target is required.

In this review, we present recent research findings in the field, including several results on the molecular mechanisms that control NASH, stress, and cell death. The correlation between the sex difference of liver functions and liver diseases and the experimental methods for NASH and liver functions in vivo are discussed.

## 2. The Mechanism of NAFLD and NASH

Dietary carbohydrates and lipids are absorbed by the small intestine and transported to the liver. The liver plays an important role in controlling energy metabolism. In a state of starvation, accumulated glucose and lipids or synthesized ketone bodies are distributed to each organ as an energy source. Conversely, in a state of energetic saturation, the liver is key in the accumulation of absorbed glucose and lipids, such as glycogen and triglycerides. In particular, a liver with a high accumulation of lipids is called fatty liver, in which numerous lipid droplets are enclosed in the hepatocytes. Fatty liver is induced by several mechanisms: (1) an increase in de novo lipogenesis from acetyl-CoA due to the upregulation of lipogenesis-related genes via transcription factors, such as SREBP1c; (2) increased uptake of fatty acids released by adipocytes into the liver; (3) suppression of β-oxidation; and (4) suppression of the release of very-low-density lipoprotein from the liver, which reduces the amount of fat in the organ. Insulin resistance, obesity, diabetes, hypertension, and dyslipidemia are known to be involved in the development of NAFLD. Nevertheless, the liver in this state has a good prognosis, with no degeneration or hepatocyte death. However, various stresses applied to the fatty liver induced liver inflammation known as NASH.

Stress responses, including oxidative stress, ER stress, and immune responses to various inflammatory cytokines, seem to be involved in the inflammation and hepatocyte death occurring during the progression of NASH. In addition, hormones secreted from the adipose tissue, the change in intestinal bacteria flora, and intestinal permeability also regulate the liver inflammation. Oxidative stress is one of the important stresses inducing inflammation in the fatty liver. The discrepancy between the generation of reactive oxygen species (ROS) and the antioxidant defenses causes oxidative stress and leads to DNA and tissue damages. Genes regulating oxidative stress such as SOD2 (the manganese-dependent superoxide dismutase) and UCP2, UCP3 (mitochondrial anion carrier proteins) are important for NASH induction and progression [9]. Mitochondrial CYP2E1 is also important for oxidative stress and cytotoxicity [10]. Mitochondrial dysfunctions, ER stress, iron metabolic dysfunctions, and gut–liver axis are involved in ROS generation and oxidative stress induction [11]. Fat and energy homeostasis in hepatocytes is controlled by mitochondrial functions such as fatty acid β-oxidation and ATP synthesis. Thus, an overload of fatty acid into mitochondria causes mitochondrial membrane dysfunction and enhances ROS generation. ER is important for assembling appropriate protein conformation. Thus, the accumulation of unfolded and misfolded proteins induces an unfolded protein response (UPR), which is regulated by the activation of UPR-related molecules (protein-kinase RNA-like ER kinase, activating transcription factor 6, and inositol requiring 1) [12]. A prolonged ER stress activates the inflammatory and cell death pathways and generates ROS through these three UPR-related signals. These activations are important for NASH progression. The excessive accumulation of iron in the liver induces oxidative stress and causes hepatocellular death [13]. Recently, a new mechanism of induction of cell death in fatty liver has been reported. Ferroptosis is an iron-dependent cell death that presents a different molecular mechanism from apoptosis and necrosis [14]. By adding an iron-chelating agent and ferroptosis inhibitors to NASH-like liver injuries in mice, cell death could be efficiently suppressed. These results indicate the importance of ferroptosis in lipotoxicity [15]. The gut–liver axis is also important for the generation of ROS and NASH progression. In both human NASH patients and animal NASH models, an increase in intestinal epithelial permeability upregulates serum endotoxin and pathogen-associated molecular patterns (PAMPs) which are inducers of hepatic inflammation [16,17]. Activation of these receptors such as the toll-like receptor 4 on liver cells induces the production of inflammatory cytokines and progresses liver inflammation and fibrosis through the activation of inflammasome [18,19]. Thus, cell junction adhesion molecules are important for the intestinal epithelial barrier function regulating NAFLD progression [20]. In addition, the NAFLD patient study revealed that the gut-microbiota dysfunction, dysbiosis, is correlated with a NASH fibrosis score [21]. The manipulation of the intestinal epithelial barrier and gut microbiota is an important candidate for a new NASH therapy.

Antioxidants protect cells from oxidation effects by free radicals and maintain redox homeostasis. Several foods and medicines contain antioxidants such as lycopine, coenzyme Q10, β-carotene, and vitamins. However, there is little evidence that these antioxidants are significantly effective for NASH therapies in vivo. Studies using vitamin E on NASH and NAFLD patients revealed that it had antioxidant and anti-inflammatory activity and that the addition of vitamin E improved the NAFLD activity score [22,23]. In contrast, vitamin E could not improve the serum liver injury-maker ALT and liver fibrosis, suggesting that the role of vitamin E for NASH treatment is not confirmed. In addition, there are some drug pipelines of novel therapies for NASH. Part of the target of these pipelines are farnesoid X receptor (FXR), peroxisome proliferator-activated receptor (PPAR), and stearoyl Co-A desaturase, which are related to lipid and cholesterol metabolism [24]. FXR is the bile acid signal receptor and OCA, 6a-ethyl chenodeoxycholic acid, is a semi-synthetic derivative of chenodeoxycholic acid, one of the major bile acids. The activation of FXR induced by OCA improves NASH pathology through multiple pathways. OCA suppresses BA formation through downregulation of CYP7A1. In addition, efflux of bile acid is induced by upregulation of BSEP, the bile acid transporter. FXR activation also regulates stellate cell activation. The study of NASH patients using OCA showed a histological improvement in NASH fibrosis [25]. The suppression of inflammation is one of the targets of NASH therapy; the chemokine receptor antagonist was considered to be a candidate for NASH treatments [26]. However, the NASH pathology is very complicated and new target molecules may be required to establish the standard therapy of NASH.

## 3. The Sex-Biased Liver Functions Regulated by B Cell Lymphoma 6

Sex-differences are found in various organs. In addition to reproductive organs, brain, liver, and other organs are known to exhibit gene expression, function, and morphological and developmental changes in males and females. NIH research funding applications now recommend allowing sufficient consideration and discussion before selecting the sex of animals used in experiments. It is no longer possible to ignore the influence of sex differences in medicine and biology. There is a need to consider both the effect of systemic sex differences such as sex hormones and the effect of local sex differences such as sex-biased gene expression in the individual organ cells.

The liver also has sex-biased functions regulated by several sex and other hormones. Sex-dependent serum growth hormone (GH) secretion rhythm from the anterior pituitary gland caused sex differences in the hepatic metabolism [27,28]. For example, drug pharmacokinetics and pharmacodynamics regulated by drug metabolic enzymes such as CYP family genes are known to differ in males and females. In humans, CYP3A activity is higher in females than in males, and melatonin, which is primarily metabolized by CYP3A, is metabolized and eliminated in vivo faster in females than in males [29]. Sex differences in the ability to metabolize xenobiotics may be involved in liver diseases such as drug-induced hepatitis and carcinogenesis. In addition, lipid metabolism enzymes can be found in the sex-different expression. Hepatic free acid uptake and esterification are increased in female livers [30]. The expression of several hepatic lipid and carbohydrate metabolic genes was different between male and female livers [31]. In addition, the phenotypes of several liver diseases differ in males and females. For example, hepatitis-derived cirrhosis and hepatocellular carcinomas are more frequent in males. One of the possible reasons for this is the protective effect of the female hormone estrogen. Estradiol inhibits the development of cirrhosis in animal models [32]. It has also been reported that estrogen suppresses the malignant transformation of hepatocytes and hepatocellular carcinoma formations by controlling the inflammatory cytokine production from Kupffer cells [33]. There are sex differences in various metabolic functions of hepatocytes, the parenchymal cells of the liver. Sex hormones circulating throughout the body regulate the sex-dependent gene expression pattern in the male and female hepatocytes [31]. In addition, clarifying the intracellular molecular mechanisms that produce functional sex differences in the liver are important for analyzing the role of sex differences in hepatic functions and liver diseases without the systemic effects of several sex hormones.

**Figure 1:**
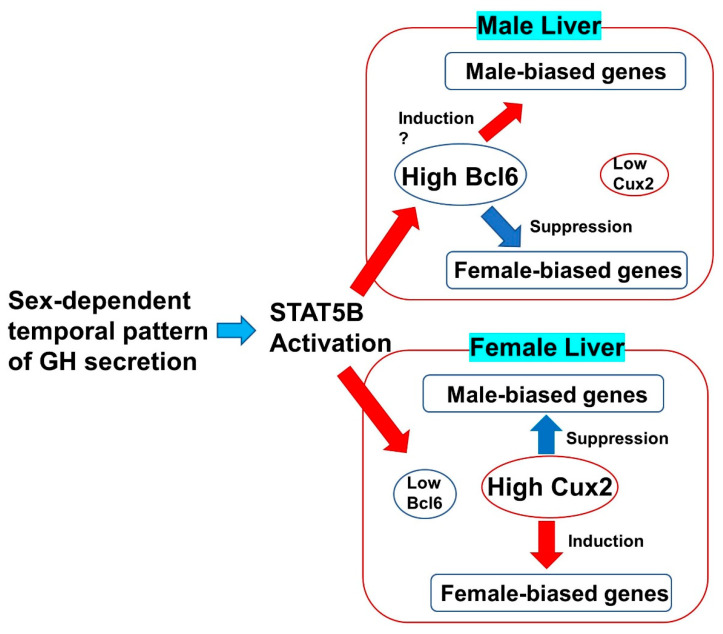
Mechanism regulating sex-biased expression in the liver. Sex differences in growth hormone (GH) serum concentration cause sex-dependent activity of Bcl6 and Cux2 in hepatocytes through the regulation of STAT5B activity. The secretion of GH from pituitary glands is regulated by steroid hormones (androgen and estrogen). These transcription factors regulate sex-biased expression of liver function genes. In addition, other sex-related transcription factors are also considered to be involved.

To identify novel molecules involved in liver function and disease, we recently conducted a comprehensive search for transcriptional regulators the expression of which changes during liver development. One of these was identified as B cell lymphoma 6 (Bcl6), a nuclear factor. Bcl6 is a key transcriptional repressor in the formation of germinal centers in the immune system, but is also involved in the development of myocarditis, as reported in the analysis of systemic gene-deficient mice [34,35]. These mice also present liver and adipose tissue atrophy and changes in the expression of fatty acid-metabolizing enzymes in the liver [36]. The studies suggest that Bcl6 plays an important role in fatty acid metabolism and in fat-related diseases in the liver. In addition, STAT5B and its downstream target Bcl6 are known to be transcriptional regulators of sex-biased genes in the liver. The mechanism regulating the serum concentration of GH differs between males and females, and this difference changes the amount of Bcl6 mRNA and protein [28]. The binding of Bcl6/STAT5 to the regulatory region of the sex-biased genes regulates the sex-based difference in liver functions [37,38]. However, Bcl6 systemic gene-deficient mice are extremely vulnerable, and most of them die approximately 5 weeks after birth [35], making it difficult to observe long-term changes in their pathological conditions. Recently, Bcl6 liver-specific deficient (Bcl6-LKO) mice crossed with Cre-expressing mice under the albumin promoter and Bcl6-Flox mice were established and the changes in the expression of sex-specific genes by Bcl6-deletion were analyzed [39,40,41]. Unlike systemic gene-deficient mice, liver-specific Bcl6 knockout mice are capable of long-term survival and Bcl6 deficiency downregulated the male-dominant CYP gene cluster, whereas the female-dominant CYP gene cluster was upregulated. A sex-dependent serum GH pattern and STAT5B activity also regulate fetal liver-biased expression of Cux2 in addition to Bcl6 [42]. This Cux2 expression is important for sex-biased liver gene expression [43]. In addition, the expression of long-noncoding RNA is controlled by the sex-biased GH and downstream factors such as Bcl6 [44]. These results suggest that GH and Bcl6 signal pathways were shown to be an important transcriptional regulator required to maintain sex differences in the male liver (Figure 1).

The next question is what mechanism regulated by Bcl6 and other transcription factors controls sex differences in the liver. Steroid hormones, especially sex hormones, play an important role in the sex-difference function of each organ in the living body. In males, testosterone is mainly secreted by the testes, binds to the androgen receptor (AR), and functions in virilizing organs such as spermatogenesis. Additionally, part of it is converted into the female hormone estrogen by the action of aromatase. In females, estrogen secreted from the ovaries induces follicle growth and ovulation, while controlling the functions of various organs. These sex hormones control the activities of the hypothalamus and pituitary glands, produce differences in serum GH secretion between male and female, and produce sex-biased gene expression in the liver through the actions of STAT5 and Bcl6 [28,45]. Androgen and estrogen receptors are present in the liver and are variably regulated by sex hormones. For example, polycystic ovary syndrome due to excess androgen causes metabolic disorders such as obesity and NASH in female livers, and hyperandrogenism has been found to be associated with the risk of developing NASH [46]. Analysis of mice with artificially elevated testosterone revealed that excessive activation of androgen receptors in the liver exacerbated hepatic steatosis, while androgen receptors were involved in regulating hepatic circadian genes in addition to fatty acid metabolism [47]. The fact that circadian clock components such as CRY1 and CRY2 control sex-biased gene expression in the liver suggests a relationship between circadian rhythms, sex hormones, and sex-biased regulation of the liver [48]. On the other hand, estrogen receptors are more strongly expressed in female livers than in male livers. Hepatocytes mainly express ERα (ESR1), and bile duct cells and stellate cells mainly express ERβ. Analysis of fatty liver and lipid metabolism in hepatocyte-specific estrogen receptor-deficient mice showed that male–female differences in fatty liver pathology are associated with estrogen effects [49].

As shown above, in addition to Bcl6, these sex steroid hormones are important for sex-biased gene expression in the liver. These sex-biased gene expressions are partly controlled by an epigenetic mechanism. The importance of epigenetic changes such as DNA methylation has been suggested as a mechanism that causes developmental and functional differences in the gene expression of several organs. The studies using mouse livers have revealed the relationship between sex-associated differentially methylated regions (sDMRs) and the expression of sex-biased genes [50]. Whole genome sequence analysis of male and female mouse livers identified that 90% of sDMRs were hypomethylated in male mice and 10% of them were hypomethylated in female mice. In addition to Bcl6, AR and ESR1 regulated sex-biased gene expression through the production and maintenance of the sex differences of sDMRs. Thus, Bcl6 and other sex-related transcription factors regulate sex-biased gene expression via the direct bindings to the enhancer and promoter regions of these genes [51].

Interestingly, liver-specific Bcl6 deletion did not show any significant changes in sex steroid hormones circulation such as β-estradiol (female hormone) and testosterone (male hormone). Furthermore, both male and female Bcl6LKO mice maintained the almost same mating ability as normal mice. These results indicate that sex differences in the liver are controlled by the transcriptional network of Bcl6 and its downstream factors, while systemic sex retains its original characteristics [41].

## 4. The Progression of NASH and Hepatocellular Carcinomas Is Regulated by Sex-Dependent Factors

These distinctions between males and females lie in the functions of various organs other than the reproductive system and are involved in several diseases. In liver diseases, there is a difference in the progression of NAFLD and NASH, which affect many younger male patients and increase in females after menopause [52]. The involvement of female hormones is considered to be the reason for this, and the effect of estradiol on liver disease and liver function has been reported [53,54]. However, the relationship between sex-dependent differences in the liver and disease remains unknown. In experiments using animal models for NAFLD, genes related to lipid and glucose metabolism have been identified as gene clusters with sex-based differences. For example, CD36 is involved in the uptake of fatty acids into muscle and adipose tissue cells, and its expression is elevated in females compared to males, suggesting that CD36 may be involved in the suppression of fatty liver development in females [55]. It is also known that the plasma glycerol level in females is higher than that in males. Aquaporin (AQP) is a selective transmembrane channel for water and glycerol, and AQP9 plays a central role in glycerol uptake in the liver and adipocytes. Expression of AQP9 in the liver changes the efficiency of glycerol uptake [56]. It has been suggested that differences in AQP between males and females may affect glucose and lipid metabolism in the liver. The expression of FRP2 differed in males and females, and FRP2 deletion induced NASH-related liver inflammatory and fibrosis in female mouse livers [57]. PPARγ expression in hepatocytes is also related to sex differences in the liver lipid accumulation and steatosis induced by a high fat diet [58]. In addition, non-parenchymal cells are also important for sex-biased liver diseases. In the mouse NAFLD model induced by a high fat diet, expression of inflammatory cytokines derived from Kupffer cells was enhanced in male livers but not in female livers [59]. These results indicate that the sex-biased gene and protein expression is important for the sex discrepancy in the progression of NAFLD and NASH. However, the sex-biased transcriptional network regulating liver diseases such as NAFLD and NASH remains unknown.

**Figure 2:**
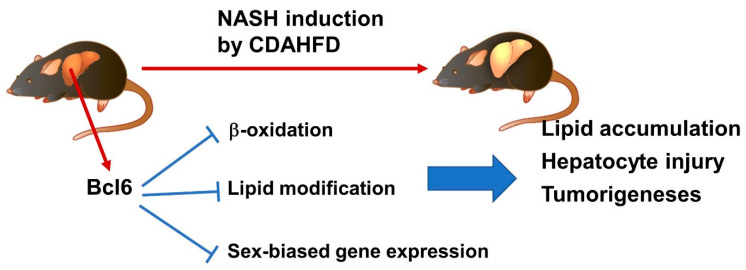
Schema of NASH regulation by Bcl6. Bcl6 regulates expression of liver functional genes such as β-oxidation, lipid modification, and sex-biased genes. In wild type livers, this genes expression is down-regulated by Bcl6 and NASH-induced lipid accumulation, injury, and tumorigenesis are progressed. In Bcl6-LKO mouse livers, up-regulation of these genes may be important for the suppression of NASH phenotypes.

As shown above, the Bcl6-LKO mouse is a model in which sex differences changed the liver only. In addition, Bcl6 systemic gene-deficient mice revealed that Bcl6 is important for lipid metabolism in the liver [36]. Bcl6 and PPARα regulate fasting-related enzyme expressions and the liver lipid accumulation is down-regulated in Bcl6-LKO mice [60]. A specific diet, a choline-deficient, L-amino acid-defined, high-fat diet (CDAHFD) was known to induce NASH-like high lipid accumulation and liver injury in mice [61]. Thus, the addition of CDAHFD to Bcl6-LKO mice revealed the effect of sex-based liver function differences on NASH pathological conditions [62]. Liver lipid accumulation, liver injury, liver fibrosis and tumorigenesis are suppressed in both female mice and Bcl6-LKO male mice, suggesting that Bcl6 is involved in the progression of NASH in this mouse model via the regulation of sex-biased gene expression or other lipid metabolic genes in the liver. Additionally, Bcl6 suppresses the β-oxidation pathway, which is involved in liver lipid accumulation [60,62]. The total and HDL cholesterol in the serum and expression of apolipoproteins C2 and E in the liver are also up-regulated in Bcl6-LKO mice [62]. Recently, the relationship between host defense in bacterial infection and liver lipid metabolism has been elucidated, and while Bcl6 has a negative effect on liver lipid accumulation and abnormal glucose tolerance, it is important for the defense against bacterial infection. In addition, it was found that ApoC3 is a Bcl6-dependent lipid regulator [63]. ApoC3 has been shown to be important for the regulation of serum TAG levels, suggesting a relationship with the NASH regulation downstream of Bcl6 [64]. It has been reported that RNA methylation is involved in sex-dependent liver lipid accumulation. The m6A methylase METTL14 in the liver regulates hepatic lipid metabolism and pathology through target RNA modification, and Bcl6 regulates the sex-dependent expression of METTL14 [65]. These studies suggested that part of the functions of Bcl6 in hepatocytes is the suppression of liver lipid β-oxidation and cholesterol and triglyceride circulation. These several multiple pathways controlled by Bcl6 may be involved in the progression of NASH and lipotoxicity in the liver (Figure 2).

## 5. Conclusions

In this review, we described stress and cell death in the liver, especially in fatty liver and steatohepatitis. With the development of therapeutic agents against the hepatitis virus, the number of patients with viral hepatitis has significantly decreased. In contrast, fatty liver associated with metabolic syndrome is becoming a serious issue as it is a major cause of liver disease. The degree of fatty acid accumulation in the liver is controlled not only by excessive calorie intake, but also by the degree of enhancement of fatty acid metabolism in the liver and its relationship with blood cholesterol and bile acid metabolism. Furthermore, in addition to lipid accumulation in the liver, several stresses, such as oxidative and ER stress, control the progression of hepatitis and hepatocytic death. Controlling such stressors and inhibiting the progression of NASH is an object for future research.

The analysis of the transcriptional regulatory system of liver sex differences is progressing. Sex differences caused by Bcl6 in bacterial infections and dyslipidemia suggested that Bcl6 is important in the evolution of immune differences between males and females [63]. In addition, nuclear receptors against sex steroid hormones are involved in sex-biased hepatic gene regulation and lipid metabolism. Estrogen, a sex hormone, has a protective effect against various inflammations and cell death in the liver, but it is still unclear how differences in metabolic liver functions in males and females effect liver disease and cell death. Gene-editing animals are used for the analyses of these metabolic functions and lipid-accumulated liver diseases. In particular, hepatic Bcl6 deletion changes the sex-biased liver gene expression while maintaining total body sex functions. Thus, Bcl6-LKO mice are a useful tool for analyzing Bcl6-downstream signals and sex differences in the liver metabolic function related to these diseases and hepatic cell death.
